# Amoxicillin Direct Oral Challenge in Hospitalized Patients With High-Risk Allergic Reactions, in a Pharmacist-Directed Program

**DOI:** 10.1093/ofid/ofag344

**Published:** 2026-06-04

**Authors:** Bryan Le, Shyam Joshi, Kendall Tucker, Young Yoon Ham

**Affiliations:** Department of Pharmacy Practice, Oregon State University College of Pharmacy, Portland, Oregon, USA; Section of Allergy and Immunology, Oregon Health & Science University, Portland, Oregon, USA; Department of Pharmacy Practice, Oregon State University College of Pharmacy, Portland, Oregon, USA; Department of Pharmacy, Oregon Health & Science University, Portland, Oregon, USA

**Keywords:** antimicrobial stewardship, direct oral challenge, high-risk reaction, inpatient evaluation, penicillin allergy

## Abstract

In 169 hospitalized adults with penicillin allergy histories, either through interview or listed in the electronic medical record of urticaria, angioedema, anaphylaxis, and/or shortness of breath at least 10 years ago, 94.1% tolerated a direct oral amoxicillin challenge led by a non-allergy specialist and were delabeled. Mild reactions occurred in 5.9%, with no severe events. This safe, effective approach may improve antibiotic stewardship, reduce costs, and support broader implementation over penicillin skin testing.

Thirty-two million people in the United States have a penicillin allergy label [1]. However, 95% of those patients with a labeled allergy are able to tolerate a penicillin upon reevaluation [2]. Tolerance of penicillins in patients with previously reported allergies may be due to reactions, even severe ones such as anaphylaxis, waning over time. In addition, misattributed family history, viral exanthem, or intolerances mistaken for allergic reaction are the most common reasons for a patient to be mislabeled [1, 2]. Labeled penicillin allergies have significant clinical and financial consequences for both patients and hospital systems. The use of alternative antibiotics results in increased risk of *Clostridioides difficile*, methicillin-resistant *Staphylococcus aureus*, and vancomycin-resistant enterococcal infections; increased odds of surgical site infections; longer length of hospital stay; and higher likelihood of antibiotic failure [1–3]. Allergy evaluation and testing plays an important role in minimizing these downsides by allowing for the use of penicillins and other β-lactams when clinically necessary. However, given the scope of the problem, there is an interest in non-allergy specialists in allergy testing and delabeling, as there are simply not enough board-certified allergists to delabel all penicillin allergies.

Penicillin allergy evaluation and testing programs are often comprised of some combination of clinical patient history, penicillin skin test (PST), and direct oral challenge (OC) [4]. PSTs are done with penicilloyl-polylysine (PRE-PEN) reagent and a dilute penicillin G solution in a 2-step process, a skin prick stage followed by an intradermal step if the skin prick testing is tolerated. An OC with amoxicillin is then used to confirm tolerance if the PST is negative [3]. While this is the standard procedure at many institutions, a cost-evaluation study in 2016 found the cost of a penicillin evaluation with PST to be at least US$220, with half of the financial burden being from the cost of PRE-PEN [5]. When patients received an OC directly, the overall cost of testing decreased to US$82 [5]. Of note, the higher cost of the PST is still cost effective compared to not evaluating the allergy and delabeling the patient, though OC is the more cost-effective method, so institutions should still institute a program with PST if that is the only option. PSTs also require specialized training, which may further limit the availability of testing, especially for hospitals with fewer resources [1]. Furthermore, while PSTs have excellent negative predictive value at 97%–99%, their positive predictive value (PPV) is less clear. There is understandably less evidence for the PPV of a failed PST, as most studies do not expose those who have not passed the PST to penicillin. Where data exist, however, the PPV ranges from 6% to 50%, which suggests that most patients who fail the skin test may well be able to tolerate a penicillin [3, 4]. Direct OC remains the gold standard for delabeling a penicillin allergy, with or without a PST [6].

## METHODS

**Figure 1.**
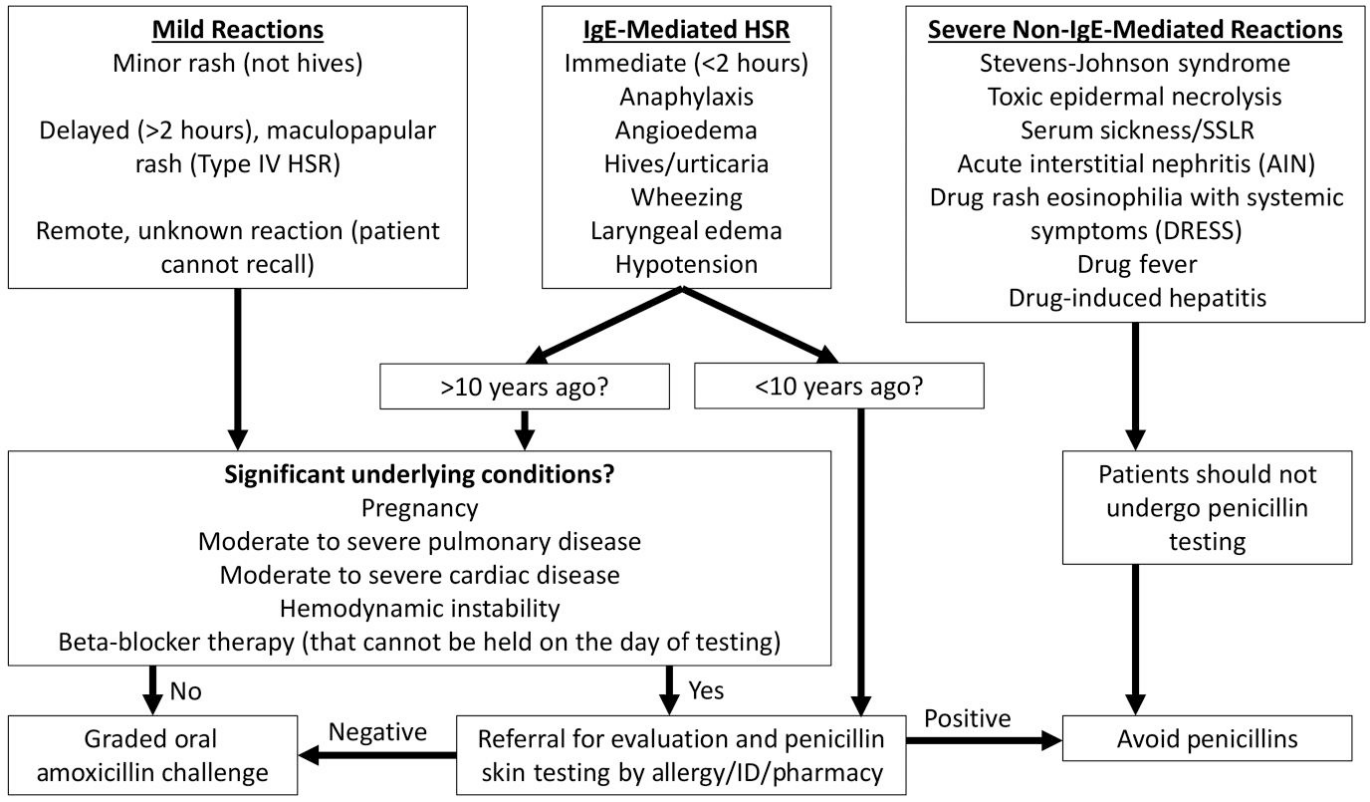
Penicillin allergy evaluation and testing algorithm. Abbreviations: AIN, acute interstitial nephritis; DRESS, drug rash eosinophilia with systemic symptoms; HSR, hypersensitivity reaction; ID, infectious diseases; IgE, immunoglobulin E; SSLR, serum sickness-like reaction.

This retrospective chart review analyzed patients with a history of a high-risk allergic reaction and who participated in an OC without a prior PST. Patients were included if they were admitted, aged >18 years at the time of the challenge, and either reported during interview or had in the allergy section of the electronic medical record a high-risk allergic reaction to a penicillin drug >10 years ago. If patients had received a penicillin since the index reaction and could be delabeled without a challenge, they were not included in the study. High-risk reactions were defined as adverse reactions with the potential to compromise the airway, such as anaphylaxis, angioedema, shortness of breath, and/or hives. Patients were excluded if they had a PST prior to the OC or a mild or unknown allergic reaction to penicillins. This study was approved by the Oregon Health & Science University institutional review board as exempt research as it was considered to be standard of care. Patients provided verbal consent, and the consent was documented in a note. At our institution, the current inpatient protocol for penicillin evaluation and testing allows for a direct OC in patients with a history of immunoglobulin E–mediated reactions such as hives, anaphylaxis, and/or angioedema, provided that the reaction happened at least 10 years ago (Figure 1). The protocol is pharmacist-driven with allergy/immunology support, with the interview, evaluation, and testing done by the pharmacist through standardized protocols, and most OCs are done on the floor; transfer to the intensive care unit (ICU) is not necessary [7]. If patients were in ICU at the time of challenge, they remained in the ICU. If patients were on the medical/surgical floor at the time of the challenge, they remained on the floor. All hospitalized patients were included.

Patients who qualified for challenge were interviewed, consented, and the OC was entered as an order set by the pharmacist after discussion with the primary team and assigned nurse. At our institution, pharmacists can be credentialed and privileged through a standardized process and are able to enter certain types of orders without provider credentials, including allergy testing. Nurses administer the amoxicillin after taking baseline vitals, and the pharmacist is paged if any reaction is reported by the patient. A specific hypersensitivity protocol for graded challenges is followed if a reaction occurs. If the patient is not able to reliably report subjective symptoms for any reason, the pharmacist remains in the room for continuous monitoring. Once the observation period is completed, the pharmacist conducts an interview with the patient to confirm that no concerning symptoms were experienced and to inform the patient of the results and next steps (eg, inform their pharmacy and outpatient providers). Once challenge is complete, a note documenting the process and results is written and the team notified. Treatment dose antibiotics can be started immediately in cases where a penicillin is the preferred choice, with orders entered by the primary team.

Our OC protocol involved 2 doses of amoxicillin suspension, 25 mg, followed 15 minutes later by 250 mg of amoxicillin, assuming no reaction to the first dose. The patients were observed for an hour following the second dose. If the patient passed the challenge without an objective reaction, the allergy would be removed from the chart. As a part of the order set, hypersensitivity reaction medications were available on an as-needed basis, including epinephrine, diphenhydramine, albuterol, and a liter of normal saline in case of hypotension. If the patient had an objective allergic reaction during observation, the OC was terminated and the penicillin allergy was not removed from the chart. The primary outcome of the study is the proportion of patients who passed a direct OC to have their penicillin allergy removed. The secondary outcomes include the number of patients who had a reaction to the OC, the type of reactions that occurred, and the number of patients who required the use of rescue medications, percentage of patients who were administered a penicillin antibiotic after OC within the first month and within the first 6 months, and delayed reactions resulting in relabeling with a penicillin allergy.

## RESULTS

**Figure 2.**
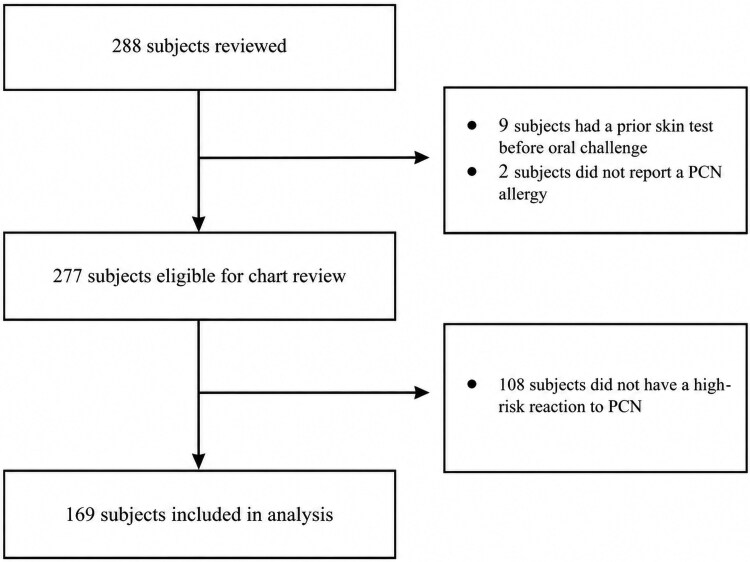
Flow diagram illustrating patient exclusion. Abbreviation: PCN, penicillin.

Two hundred eighty-eight patients with a penicillin drug allergy label underwent an OC from September 2020 to February 2024. Nine patients were excluded due to a prior PST and 2 were excluded because they did not endorse a penicillin allergy, though they were challenged due to a prior reaction to cephalexin and concern about reaction to the amino side chain of amoxicillin. One hundred eight patients were excluded due to their index reaction being low risk (eg, indeterminate rash, unknown reaction, or delayed rash). A total of 169 patients were included in the primary analysis (Figure 2).

The baseline characteristics for the patient populations are outlined in Table 1. The median age of participants was 61 years, 55.6% were female, and 84.6% were White. Most patients reported an allergy to a generic penicillin (89.3%) while a minority mentioned a specific penicillin such as amoxicillin or ampicillin (33.7%) or piperacillin-tazobactam (1.2%). For the index event, 27.2% and 20.1% had a historical reaction of anaphylaxis or angioedema to penicillin drugs, respectively, while 8.9% reported shortness of breath or dyspnea and 54.4% reported a reaction of hives. As patients sometimes reported multiple high-risk symptoms, the percentages sum to more than 100%. If a patient reported a constellation of symptoms that would indicate anaphylaxis but did not report anaphylaxis specifically, they are not included as having an index reaction of anaphylaxis.

**Table 1.** Baseline Patient Characteristics

Characteristic	Patients (N = 169)
Median age, y	61
Sex, female	94 (55.6)
Race, White	143 (84.6)
PCN allergy listed	
PCN or unspecified PCN	151 (89.3)
Amoxicillin or ampicillin	57 (33.7)
Piperacillin/tazobactam	2 (1.2)
Index event	
Anaphylaxis	46 (27.2)
Angioedema	34 (20.1)
Hives	92 (54.4)

Data are presented as No. (%) unless otherwise indicated.

Abbreviation: PCN, penicillin.

**Figure 3.**
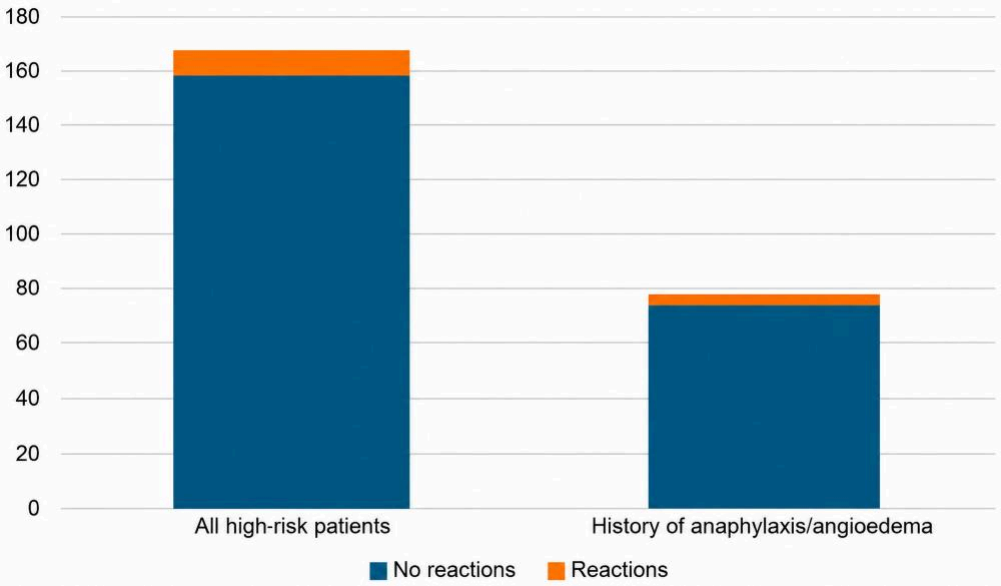
Results of direct oral challenge. All high-risk patients included participants who endorsed an allergic response to penicillin products that consisted of anaphylaxis, angioedema, shortness of breath, and/or urticaria. Participants who explicitly described having an event closely aligned to anaphylaxis and/or angioedema from the use of penicillin products were included in a separate group labeled as “history of anaphylaxis/angioedema.”

One hundred fifty-nine patients (94.1%) tolerated the OC without any concerning symptoms and the allergy was removed. For patients who had an index reaction of either anaphylaxis or angioedema, 75 of 80 (93.8%) tolerated the OC and had their allergy delabeled (Figure 3). The 5 patients with a history of anaphylaxis and/or angioedema who reported symptoms described lip tingling, itching, light-headedness, and facial flushing. Most of the symptoms were subjective and their vitals remained unchanged throughout the observation period. One of those 5 patients received intravenous diphenhydramine to relieve subjective itching. The other 5 patients who did not pass the OC had an index reaction of hives or dyspnea. Of the 10 patients overall (5.9%) who reacted during the OC, 3 required a single dose of 25 mg intravenous diphenhydramine and 1 patient received albuterol due to shortness of breath. Epinephrine was not used for any patient (Table 2). The majority of patients who reacted only needed to be observed as their reaction subsided. Fifty-one percent of those who passed the challenge were started on a penicillin antibiotic within the first month postevaluation (eg, ampicillin, piperacillin-tazobactam, amoxicillin, amoxicillin-clavulanate), while 67% were started on a penicillin antibiotic within the first year. Two delabeled patients were relabeled due to potential delayed reactions. One patient had a mild delayed reaction of maculopapular rash, while the other reported itching in her finger 1 week after OC and requested that penicillin be added back to her allergy list. Epinephrine was not utilized for either reaction and neither was consistent with a severe cutaneous adverse reaction.

**Table 2.** Safety of Direct Oral Challenge

Results	Patients Who Reacted (n = 10)
Reaction	
Gastrointestinal symptoms^a^	2 (20)
Pruritis^b^	4 (40)
Urticaria	1 (10)
Shortness of breath	1 (10)
Other reactions	5 (50)
Use of rescue medication	
Diphenhydramine	3 (30)
Albuterol	1 (10)
None/Observation	6 (60)

Data are presented as No. (%).

Patients treated through the penicillin oral challenge were first treated with amoxicillin suspension 25 mg followed by amoxicillin suspension 250 mg assuming no reaction to the first dose.

^a^Gastrointestinal symptoms included nausea, vomiting, diarrhea, and/or stomach upset.

^b^Pruritus was recorded as a subjective finding endorsed by the patient.

## DISCUSSION

Recently, studies have been conducted examining the safety of direct OCs in patients without a prior PST [6, 8]. The PALACE trial was a randomized clinical trial that compared direct OC to standard of care skin testing followed by an oral challenge. The study focused on patients with low-risk reactions (PEN-FAST score of <3 was included, though most patients had a score of 0 or 1) and found that direct OC was noninferior to standard of care. No serious adverse events occurred [6]. Multiple retrospective studies have tested patients with low-risk penicillin allergies by bypassing PST, all with a high degree of success (>95% of patients delabeled) [8–11]. Missing from these studies, however, is direct OC of patients with a history of higher-risk reactions, such as hives, angioedema, and anaphylaxis [6, 11, 12]. This study expands upon these findings by only evaluating patients at risk of respiratory compromise, with more than half of patients reporting a history of anaphylaxis or angioedema.

Direct OC without a prior PST for patients who report a high-risk allergic reaction to penicillin drugs appears to be a reasonably safe way of evaluating patients once 10 years have passed from the initial reaction. OC without a prior PST can be more cost effective and easier to administer, requiring fewer resources. It can also be administered without additional training, which is required for PST. Our study demonstrates the utility of a pharmacist-directed delabeling protocol using direct OC. Our study is limited in that it is single center and has considerable resources as an academic medical center. In addition, the original reactions as reported by the patients are temporally distant from the evaluation and challenge and may not accurately reflect the index reaction. It is possible that a more objective assessment of the original reaction may find higher reaction rates when the index reaction was supported by the medical record beyond patient interview. More studies are needed to fully assess the safety and efficacy of OC in the setting of high-risk reactions, but our experience thus far has been promising. Given the impact of penicillin allergies in clinical outcomes and ability to use preferred antibiotics, other systems should consider adding such a protocol to their armamentarium to optimize the care of patients.
